# Ubiquitin-Specific Peptidase 5, a Target Molecule of Vialinin A, Is a Key Molecule of TNF-α Production in RBL-2H3 Cells

**DOI:** 10.1371/journal.pone.0080931

**Published:** 2013-12-04

**Authors:** Yasukiyo Yoshioka, Yue Qi Ye, Kiyoshi Okada, Kayoko Taniguchi, Ayaka Yoshida, Kouichi Sugaya, Jun-ichi Onose, Hiroyuki Koshino, Shunya Takahashi, Arata Yajima, Shunsuke Yajima, Naoki Abe

**Affiliations:** 1 Department of Nutritional Science, Faculty of Applied Bio-science, Tokyo University of Agriculture, Tokyo, Japan; 2 Department of Bioscience, Faculty of Applied Bio-science, Tokyo University of Agriculture, Tokyo, Japan; 3 Department of Fermentation Science, Faculty of Applied Bio-science, Tokyo University of Agriculture, Tokyo, Japan; 4 RIKEN (The Institute of Physical and Chemical Research), Wako, Saitama, Japan; Stanford University, United States of America

## Abstract

Tumor necrosis factor alpha (TNF-α), a central mediator of the inflammatory response, is released from basophilic cells and other cells in response to a variety of proinflammatory stimuli. Vialinin A is a potent inhibitor of TNF-α production and is released from RBL-2H3 cells. Ubiquitin-specific peptidase 5 (USP5), a deubiquitinating enzyme, was identified as a target molecule of vialinin A and its enzymatic activity was inhibited by vialinin A. Here we report production of TNF-α is decreased in USP5 siRNA-knockdown RBL-2H3 cells, compared with control cells. The finding of the present study strongly suggests that USP5 is one of the essential molecules for the production of TNF-α in RBL-2H3.

## Introduction

Mast cells play a central role in allergic responses by secreting various inflammatory mediators [1], [2]. Upon activation by high-affinity IgE receptors, mast cells release factors, such as histamine, cytokines, and chemokines that ultimately cause allergic responses [3], [4]. Mast cells secrete inflammatory cytokines such as tumor necrosis factor-α (TNF-α) and interleukin-4 (IL-4) that are produced by the activation of transcription factors upon stimulation [5], [6]. In particular, dysregulated TNF-α production and release are implicated in a wide range of inflammatory diseases such as rheumatoid arthritis and Crohn's disease. Thus, drugs that selectively target TNF-α in activated mast cells and basophils are promising therapeutic candidates for rheumatoid arthritis and Crohn's disease.

The ubiquitin pathway is necessary throughout all stages of eukaryotic cell development. The dynamic modification of a substrate protein with ubiquitin can modify its function, localization and fate in the cell [7]. Ubiquitin conjugation relies on a cascade of enzymes, and its removal is mediated by deubiquitinating enzymes (DUBs), the majority of which are cysteine proteases. Understanding the function of ubiquitin hydrolase in immunology and infection has attracted increasing interest due in part to their discovery in systems that lack endogenous ubiquitin/proteasome machinery [8]–[10].

We previously reported that vialinin A was a strong 2,3-diphenyl-1-picrylhydrazyl free radical-scavenger that could be isolated from the dry fruiting bodies of an edible Chinese mushroom, *Thelephora vialis* [11] and potently inhibited TNF-α release from antigen-stimulated rat basophilic leukemia (RBL-2H3) cells with an IC_50_ of 0.09 nM and murine bone marrow-derived mast cells with an IC_50_ of 0.04 nM [12], [13]. Moreover, vialinin A inhibited the release of TNF-α in a dose dependent manner, while this compound inhibited TNF-α production at low concentrations without a dose-dependency [13]. This observation suggested that vialinin A could have respective operating points for TNF-α production and release in RBL-2H3.

RBL-2H3, which has the phenotypic characteristics of mucosal mast cells, is a tumor analog of mast cells widely used in mast cell-associated studies. After antigen stimulation, these cells release β-hexosaminidase, a marker of mast cell degranulation, and inflammatory cytokines [14]. In subsequent studies, we identified a DUB, ubiquitin-specific peptidase 5/isopeptidase T (USP5/IsoT, EC3.1.2.15), as a target molecule of vialinin A in RBL-2H3 cells, and vialinin A inhibited the USP5/IsoT activity *in vitro* [15].

In the present study, we investigated the TNF-α and β-hexosaminidase release from DUB knockdown cells. Moreover the correlation between the inhibitory effect of antigen-induced TNF-α production in and release from RBL-2H3 cells and the suppressive effects of USP5 gene expression in the cells was demonstrated.

## Materials and Methods

### RNA

The synthesized oligonucleotides (Invitrogen, Carlsbad, CA) targeting USP4, USP5, USP13, and a non-targeting negative control are listed in Table S1.

### Cell culture, transfection and treatment

RBL-2H3 cells [16] were cultured in Dulbecco's modified Eagle's medium (Nissui Pharmaceutical Co., Tokyo, Japan) containing 10% (v/v) fetal bovine serum (Gibco Life Technologies, Grand Island, NY) in an incubator with 5% CO_2_ at 37°C. At confluency, cells were resuspended in fresh medium and transfected with siRNA at a final concentration of 20 nM using Lipofectamine RNAiMAX transfection reagent (Invitrogen) according to the manufacturer's instructions [17], [18]. Cells transfected with non-target siRNA were used as a negative control. Cells receiving Dulbecco's modified Eagle's medium without any RNA served as mock-transfected control cells. Six hours after transfection, all cells (siRNA transfection control, non-target siRNA control, and mock transfection control cells) were sensitized with 200 ng/mL DNP-specific IgE (Sigma) for 16 h. The cells were challenged with 20 ng/mL DNP-bovine serum albumin (BSA; Cosmo Bio Co. Ltd., Tokyo, Japan) at 37°C for 1 h (TNF-α mRNA) or 3 h (TNF-α protein) before proceeding to the subsequent experiments. All experiments were performed in triplicates.

### Reverse transcription-polymerase chain reaction (RT-PCR)

For quantitative analysis of gene expression by real-time PCR, total RNA was isolated from cell culture lysates according to the RNeasy protocol (Qiagen, Hilden, Germany). USP4, USP5, and USP13 were detected from antigen-stimulated RBL-2H3 cells transfected with each siRNA. RNA purity was confirmed by measuring the 260/280 nm absorbance ratio and RNA concentrations were quantified by measuring the optical density at 260 nm using a U-0080D spectrophotometer (HITACHI, Tokyo, Japan). The primer sequences used in the experiments are listed in Table S2. The purified RNA was reverse-transcribed by TaqMan Reverse Transcription reagent (Applied Biosystems, Carlsbad, CA) according to the manufacturer's protocol. The real-time PCR reactions were performed using a Power SYBR Green PCR Master Mix (Applied Biosystems) and the thermal conditions for denaturation, annealing and extension are listed in Table S3. For each sample, mRNA expression was normalized to the control value that was measured for glyceraldehyde 3-phosphate dehydrogenase (GAPDH). PCR products were also resolved in 2.0% agarose gels, stained with ethidium bromide, and photographed under ultraviolet light. Detection and densitometric analyses of bands were performed using an Image Quant 400 (GE Healthcare, Tokyo, Japan). Values were normalized to GAPDH and expressed as relative expression levels.

### Western blot analysis

Intracellular proteins were extracted with 0.2%Triton X-100 lysis buffer containing protein inhibitor cocktail from antigen-stimulated RBL-2H3 cells transfected with each siRNA. Protein samples were treated with SDS sample buffer containing with 2% SDS and 5% β-mercaptoethanol at 100°C for 3 min. Samples were separated by SDS-polyacrylamide gel electrophoresis and transferred to a polyvinylidene fluoride membrane. Western blotting for USP4, the membrane was blocked in 0.4% gelatin-phosphate-buffered-saline for 30 min at room temperature and a rabbit anti-USP4 antibody (1∶250 dilution) and an anti-GAPDH antibody purchased from Santa Cruz Biotechnology, Inc. (Santa Cruz, CA), respectively, was used for overnight at 4°C. Western blotting for USP5 and USP13, the membrane was blocked in 0.05% casein-phosphate-buffered-saline for 60 min at room temperature and a rabbit anti-USP5 antibody (1∶250 dilution) purchased from Abgent (San Diego, CA) or an anti-USP13 antibody (1∶500 dilution) obtained from Protein Tech Group (Chicago, IL) was used for overnight at 4°C. A goat anti-rabbit IgG conjugated with horseradish peroxidase was used as a secondary antibody, and detection was performed using a colorimetric substrate 3,3′-diaminobenzidine tetrahydrochloride. Densitometric analysis was performed with Image Quant 400.

### Degranulation assay and measurement of released TNF-α and IL-4

Degranulation of RBL-2H3 cells was monitored by measuring β-hexosaminidase activity. The medium of siRNA and IgE-antigen cells treated as described above was collected and the level of β-hexosaminidase that was released into the medium was determined using a colorimetric assay with *p*-nitrophenyl-*N*-acetyl-β-D-glucosaminide (Calbiochem, La Jolla, CA). β-Hexosaminidase release was expressed as the percentage of activity of released β-hexosaminidase relative to that of mock-transfected cells. Using the same media, the total amount of TNF-α that was released from and remained in the cells was determined using the rat Quantikine TNF-α enzyme-linked immunosorbent assay (ELISA) kit (R&D Systems, Minneapolis, MN) and rat Quantikine IL-4 ELISA kit (R & D). The amount of released TNF-α was determined to measure extracellular TNF-α with ELISA. TNF-α production was evaluated to measure the total amount of the extracellular TNF-α, which was released outside the cells, and the intracellular one, which remained inside the cells.

### Cytotoxicity assay

The cytotoxicity of each siRNA knockdown for RBL-2H3 cells was determined by measuring the release of lactate dehydrogenase, a cytoplasmic enzyme. Lysis with 0.2% Triton X-100 was performed to quantify the lactate dehydrogenase remaining in cells. The lactate dehydrogenase level in both the supernatant and lysate were assayed using an LDH assay kit (Promega, Madison, WI). The total amount of lactate dehydrogenase from both the supernatant and lysate was taken as 100%.

### Statistical analysis

Numerical data are expressed as mean ± standard deviation of the mean. Differences were evaluated using Student's *t*-test, and values of *p*<0.05 and *p*<0.1 were considered statistically significant.

## Results

### siRNA-based gene knockdown of DUBs in RBL-2H3 cells

**Figure 1:**
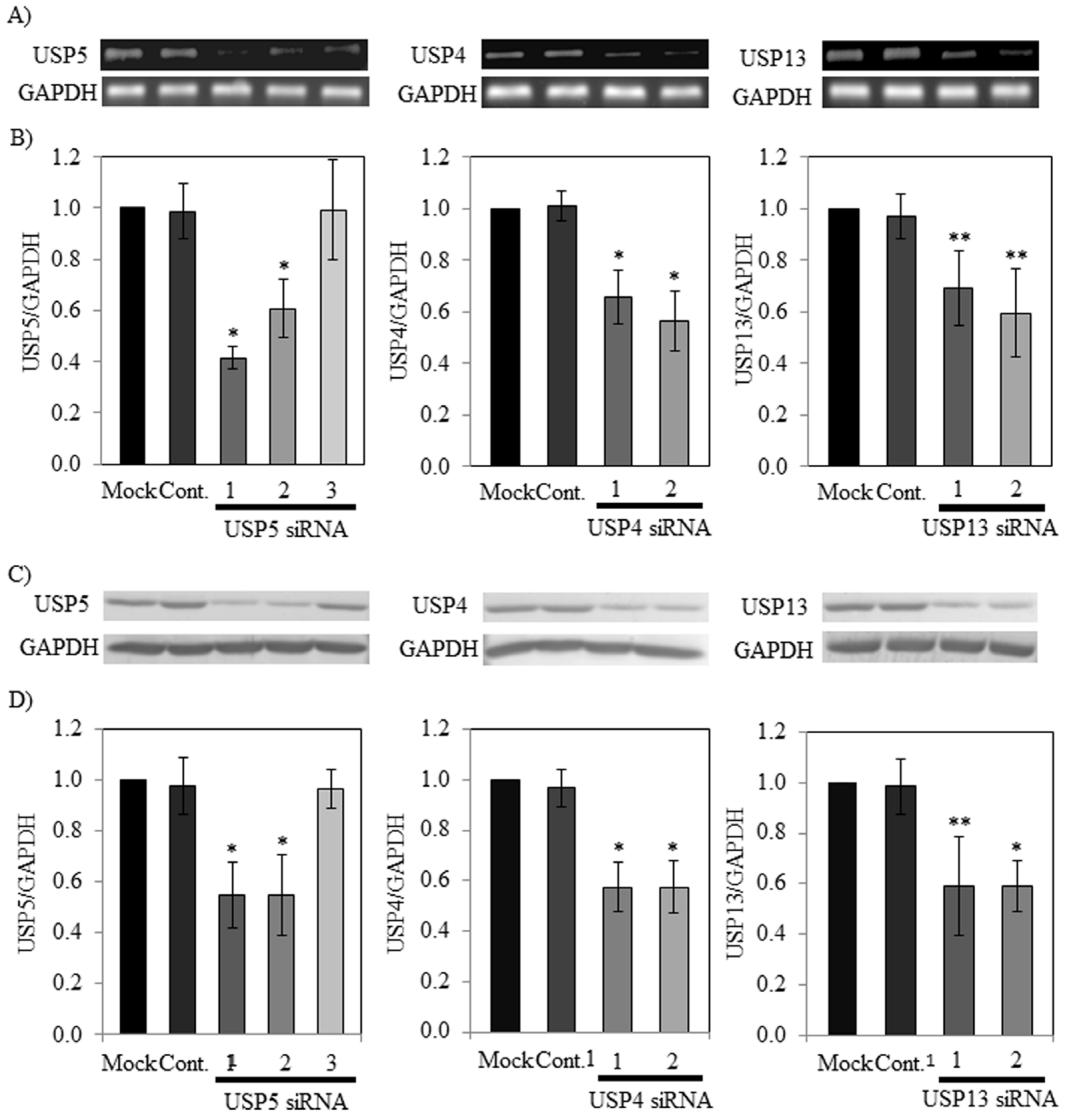
Expression of DUB mRNAs and protein level of DUBs in DUB siRNA knockdown cells. (A) Expression of USP4, USP5, and USP13 mRNAs in RBL-2H3 cells treated with each DUB siRNA was analyzed by real-time RT-PCR. PCR products were detected with agarose gel electrophoresis. (B) Band intensities of PCR products were measured and compared relative to GAPDH. The GAPDH expression pattern in RBL-2H3 cells was used as an internal control. (C) Protein level of USP4, USP5, and USP13 in RBL-2H3 cells treated with each DUB siRNA was analyzed by western blotting. (D) Intensities of detected bands were measured and compared relative to GAPDH. The GAPDH level pattern in RBL-2H3 cells was used as an internal control. Each value represents mean ± standard deviation of triplicate determinations. The significance of differences from each control value was calculated using the Student's t-test (*p<0.05, **p<0.1).

To investigate the suppressive effects of siRNAs against the gene expression of USP4, USP5, and USP13 in RBL-2H3 cells, we used siRNA-based gene knockdown of these DUBs by using 2 or 3 positions as siRNA targets for each DUB. As the results of real-time PCR and western blotting analyses, all the DUB siRNAs except for USP5 siRNA3 significantly decreased both expression levels of the mRNA and the protein of the target DUB in each siRNA-transfected RBL-2H3 compared with those in the control siRNA- and mock-transfected cells (Figure 1). The lactate dehydrogenase activities of all supernatants in the siRNA-based gene knockdown cells were below 5%, indicating that these RNAi transfections did not make effects (Figure S3).

### Effects of DUB siRNAs on degranulation from transfected-RBL-2H3 cells

As an indicator of the degranulation from RBL-2H3 cells, the release of β-hexosaminidase into the supernatant was measured. When each of the siRNA-transfected RBL-2H3 cells was stimulated with DNP-BSA, all RNAi experiments revealed no change in the β-hexosaminidase level (Figure S1).

### Effect of DUB siRNAs on TNF-α and IL-4 production in and release from transfected-RBL-2H3 cells

**Figure 2:**
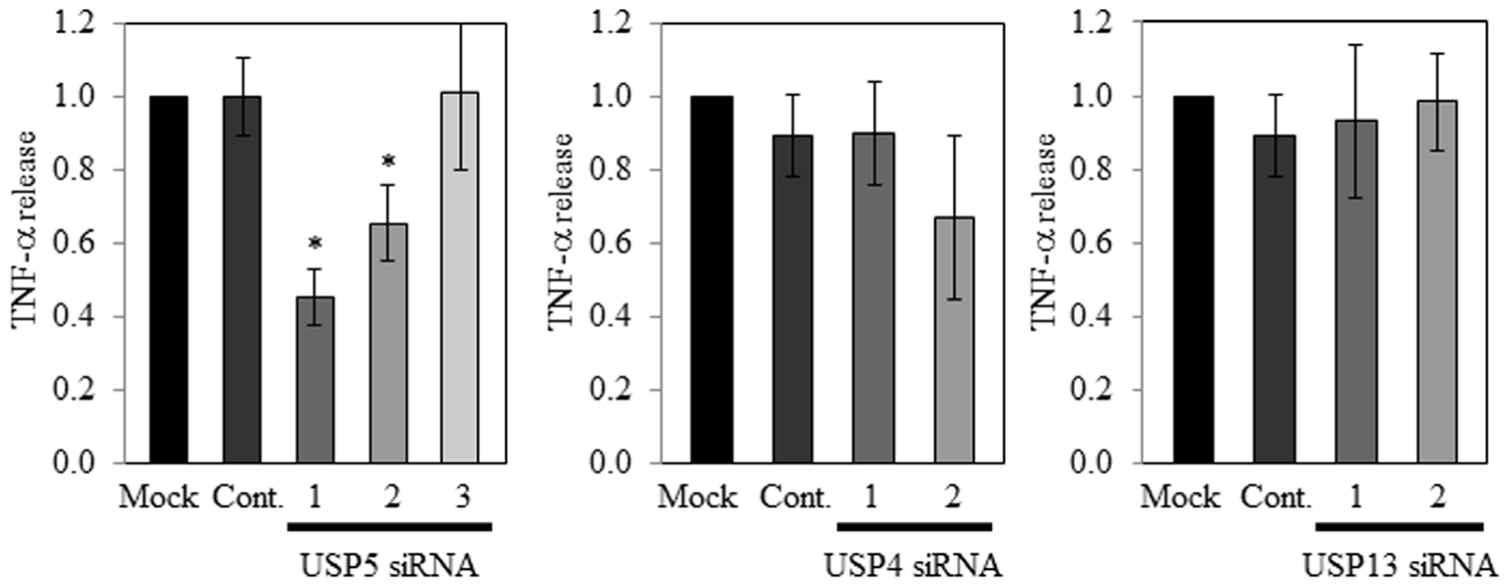
TNF-α release from DUB siRNA-knockdown cells. Amounts of TNF-α and from RBL-2H3 cells treated by DUB siRNA were determined by ELISA. Each value represents mean ± standard deviation of triplicate determinations. The significance of differences from each control value was calculated using the Student's t-test (*p<0.05).

**Figure 3:**
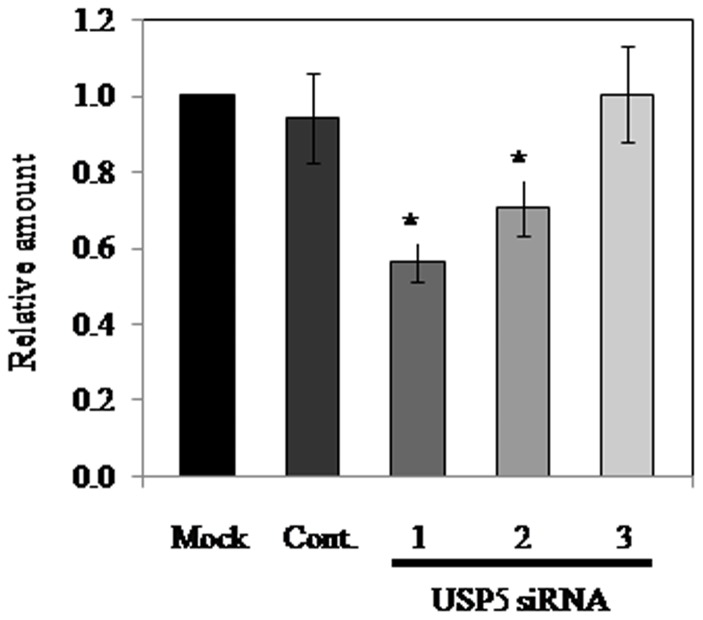
TNF-α production in USP5 siRNA-knockdown cells. TNF-α production was calculated as the total amount of TNF-α released into the supernatant and TNF-α remaining within the cells based on ELISA. The significance of differences from control values was calculated using the Student's t-test (*p<0.05).

We have found that vialinin A could inhibit TNF-α release [12] and the enzymatic activities of USP5 [15]. In addition, we reported that vialinin A might inhibit TNF-α production and release at respective operating points in RBL-2H3 [13]. Therefore, the effects of DUB siRNAs on the TNF-α release from the cells were examined, followed by measuring TNF-α production in the cells. In the RNAi experiments for USP4 and USP13, no significant inhibition of TNF-α release was observed for either of these DUBs (Figure 2). On the other hand, USP5 siRNA1 and USP5 siRNA2 resulted in significant suppression of both of TNF-α production in and release from each siRNA-transfected RBL-2H3 in a dose dependent manner (Figures 2 and 3). We have also found that vialinin A could inhibit IL-4 release [12]. No effect on IL-4 release from siRNA transfected RBL-2H3 cells compared with control cells was observed (Figure S2).

### Effect on expression of TNF-α mRNA by USP5 siRNAs in transfected-RBL-2H3 cells

**Figure 4:**
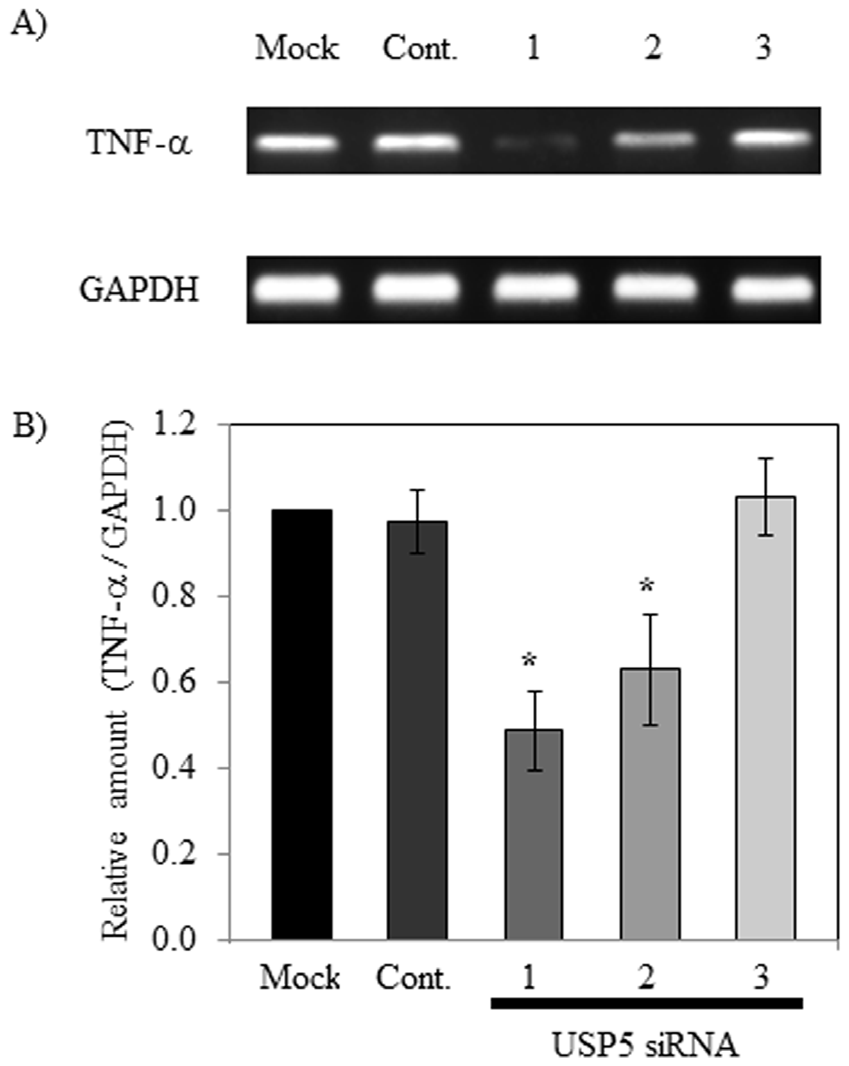
Expression of TNF-α mRNA in USP5 siRNA-knockdown cells. (A) Expression of TNF-α mRNA in RBL-2H3 cells treated with USP5 siRNAs were analyzed by real-time RT-PCR. PCR products were detected with agarose gel electrophoresis. (B) Band intensities of PCR products were measured and compared to GAPDH. The GAPDH expression pattern in RBL-2H3 cells was used as an internal control. The significance of differences from control values was calculated using the Student's t-test (*p<0.05).

Because inhibition of TNF-α production in RBL-2H3 cells treated with USP5 siRNA1 and USP5 siRNA2 was observed (Figure 3), we examined the effect of USP5 siRNA1 and USP5 siRNA2 on TNF-α mRNA expression. Treatment with USP5 siRNA1 and USP5 siRNA2 significantly suppressed TNF-α mRNA expression in RBL-2H3 cells (Figure 4).

## Discussion

We previously reported that vialinin A could dose-dependently inhibit TNF-α release [12] and the enzymatic activities of USP5 [15]. Further investigation of inhibitory effects of vialinin A demonstrated that, unlike the inhibition of TNF-α release, vialinin A inhibited TNF-α production at low concentrations without a dose-dependency [13]. This observation suggested that vialinin A could have the respective operating points for TNF-α production and release. Therefore, in the present study, we examined whether USP5 could control the production and/or release of TNF-α from RBL-2H3 cells. We knocked down USP5 with siRNA by using 3 positions as siRNA targets. USP5 siRNA1 and siRNA2 significantly decreased both expression levels of the mRNA and the protein of USP5 (Figure 1). Furthermore, the specific loss of USP5 mRNA led to a 40% to 50% decline in the level of TNF-α release, as measured by ELISA (Figure 2). We previously reported that vialinin A inhibits the release of TNF-α, but not that of β-hexosaminidase, which is commonly used as a degranulation marker for chemokines, such as histamine [12]. This finding was in contrast to the reported action of tacrolimus, which can potently suppress both TNF-α and β-hexosaminidase release. Because our RNAi experiments demonstrated no changes in the level of β-hexosaminidase release (Figure S1), the modulation of USP5 expression by the siRNAs may be in parallel to the effect of vialinin A. Although vialinin A was reported to inhibit IL-4 release [12], no effect of DUB siRNAs on IL-4 release was observed (Figure S2). Thus, it was suggested that vialinin A have different inhibition point(s) against the production of TNF-α and that of IL-4 in RBL-2H3 cells.

We also examined other DUBs, USP4 and USP13, in RNAi experiments. No significant inhibition of TNF-α release by these DUBs was observed. This is in contrast to the effect on enzymatic activity, where USP4 was strongly inhibited by vialinin A. Although USP13 is a homolog of USP5, these results might be due to a different interaction pattern than that with other proteins [19], [20].

Our findings suggested that the inhibitory mechanism of TNF-α production by vialinin A may be different from those of known inhibitors such as tacrolimus. A transcription factor NF-κB is known as the key regulator of TNF-α mRNA transcription [21], and NF-κB activation through degradation of IκB is known to be regulated by the combination of ubiquitination and deubiquitination [22], namely, degradation of IκB polyubiquitinated at K48 in the proteasome can activate NF-κB. Because USP5 degrades unanchored polyubiquitin chains to free ubiquitin [23], [24], inhibition of the USP5 enzymatic activity in RBL-2H3 cells should cause the accumulation of unanchored polyubiquitin chains which compete with substrates for 26S proteasome binding [25], [26]. That is to say, because expression of TNF-α mRNA is decreased in USP5 knockdown cells, vialinin A may indirectly inhibit TNF-α production through suppression of NF-κB activation caused by accumulation of unanchored polyubiquitin chains which results in inhibition of IκB degradation.

The findings of the present study demonstrated that USP5, a target molecule of vialinin A, mightbe a key molecule for the production of TNF-α. Therefore, the findings regarding USP5 may be good starting points for clarification of the inhibitory mechanism of vialinin A against TNF-α production and release and, subsequently, the future development of anti-TNF-α therapies.

## Supporting Information

Figure S1 **Effect on β-hexosaminidase release from DUB siRNA-knockdown cells.** Amounts of β-hexosaminidase release from RBL-2H3 cells treated by DUB siRNA were determined using a colorimetric assay with p-nitrophenyl-N-acetyl-β-D-glucosaminide.
(TIF)

Figure S2 **Effect on IL-4 release from DUB siRNA-knockdown cells.** Amount of IL-4 release from RBL-2H3 cells treated by DUB siRNA were determined by ELISA.
(TIF)

Figure S3 **Cell viability for DUB siRNA-knockdown cells.** Effect on lactate dehydrogenase leakage from RBL-2H3 cells treated by DUB siRNA. Amount of lactate dehydrogenase were determined by measuring absorbance 490 nm.
(TIF)

Table S1 **siRNA sequences.**
(TIF)

Table S2 **Primers used for real-time PCR.**
(TIF)

Table S3 **Conditions used for real-time PCR.**
(TIF)
